# Adaptive clinical trial designs for European marketing authorization: a survey of scientific advice letters from the European Medicines Agency

**DOI:** 10.1186/1745-6215-15-383

**Published:** 2014-10-02

**Authors:** Amelie Elsäßer, Jan Regnstrom, Thorsten Vetter, Franz Koenig, Robert James Hemmings, Martina Greco, Marisa Papaluca-Amati, Martin Posch

**Affiliations:** Institute of Medical Biostatistics, Epidemiology and Informatics, University Medical Center Mainz, Obere Zahlbacher Str. 69, 55131 Mainz, Germany; European Medicines Agency, 30 Churchill Place, London, E14 5EU UK; Center for Medical Statistics, Informatics and Intelligent Systems, Medical University of Vienna, Spitalgasse 23, 1090 Vienna, Austria; Medicines and Healthcare Products Regulatory Agency, 151 Buckingham Palace Road, London, SW1W 9SZ UK

**Keywords:** adaptive design, EMA, FDA, seamless designs, scientific advice, clinical trials, phase II-III, pivotal trial, orphan drugs

## Abstract

**Background:**

Since the first methodological publications on adaptive study design approaches in the 1990s, the application of these approaches in drug development has raised increasing interest among academia, industry and regulators. The European Medicines Agency (EMA) as well as the Food and Drug Administration (FDA) have published guidance documents addressing the potentials and limitations of adaptive designs in the regulatory context. Since there is limited experience in the implementation and interpretation of adaptive clinical trials, early interaction with regulators is recommended. The EMA offers such interactions through scientific advice and protocol assistance procedures.

**Methods:**

We performed a text search of scientific advice letters issued between 1 January 2007 and 8 May 2012 that contained relevant key terms. Letters containing questions related to adaptive clinical trials in phases II or III were selected for further analysis. From the selected letters, important characteristics of the proposed design and its context in the drug development program, as well as the responses of the Committee for Human Medicinal Products (CHMP)/Scientific Advice Working Party (SAWP), were extracted and categorized. For 41 more recent procedures (1 January 2009 to 8 May 2012), additional details of the trial design and the CHMP/SAWP responses were assessed. In addition, case studies are presented as examples.

**Results:**

Over a range of 5½ years, 59 scientific advices were identified that address adaptive study designs in phase II and phase III clinical trials. Almost all were proposed as confirmatory phase III or phase II/III studies. The most frequently proposed adaptation was sample size reassessment, followed by dropping of treatment arms and population enrichment. While 12 (20%) of the 59 proposals for an adaptive clinical trial were not accepted, the great majority of proposals were accepted (15, 25%) or conditionally accepted (32, 54%). In the more recent 41 procedures, the most frequent concerns raised by CHMP/SAWP were insufficient justifications of the adaptation strategy, type I error rate control and bias.

**Conclusions:**

For the majority of proposed adaptive clinical trials, an overall positive opinion was given albeit with critical comments. Type I error rate control, bias and the justification of the design are common issues raised by the CHMP/SAWP.

## Background

While the development of statistical methods for the design and analysis of adaptive clinical trials began more than 20 years ago [1, 2], there is still limited experience in the application of such designs in clinical trials. Following a workshop at the European Medicines Agency (EMA) involving academia, industry and regulators in 2007, the *Reflection Paper on Methodological Issues in Confirmatory Clinical Trials Planned with an Adaptive Design* (CHMP/EWP/2459/02) [3] was published. The US Food and Drug Administration (FDA) followed in 2010, publishing draft guidance on adaptive design clinical trials for drugs and biologics [4]. Because many of the designs proposed are nonstandard and address the particular application being considered, and because the experience of sponsors as well as regulators in planning, conducting and interpreting clinical trials applying adaptive designs is limited, early interaction with regulators is important. EMA offers developers of medicinal products scientific advice (SA) and protocol assistance (PA) as formal procedures for such interactions. Protocol assistance denotes the process of providing scientific advice for designated orphan drugs. For simplicity, in the remainder of this paper, both procedures (SA and PA) will both be referred to as scientific advice. SA is provided by the Committee for Human Medicinal Products (CHMP) through the Scientific Advice Working Party (SAWP). In the European Union, it is not mandatory for sponsors of medicinal products to request SA, and SA is not legally binding with regard to any future marketing authorization application of the product concerned, either for the regulatory agency, or for the sponsor. SA can be requested on all scientific aspects of drug development (quality, nonclinical and/or clinical issues), including methodological and statistical issues, during the initial development of a medicinal product, as well as later on, during the post-authorization phase. Sponsors may ask for ‘follow-up’ to the initial request for SA. The current SA procedure is streamlined to allow finalization within 40 or maximally 70 days and includes involvement of the CHMP by a formalized peer review before final adoption of the SA letter. Adherence by the sponsor to SA provided by the CHMP/SAWP has been identified as a predictor of positive outcome of a marketing authorization application for pharmaceutical drugs submitted to the EMA [5].

Since 2007 a growing number of scientific advice procedures relating to adaptive study designs were requested by sponsors. This observation is consistent with a recent survey by Morgan *et al*. [6] that noted an increase in the use of more complex adaptive designs for which early engagement with regulators is encouraged. The purpose of this survey is to provide an overview on the type of adaptive designs proposed and the issues that were identified during the procedures. We assessed the settings in which adaptive designs were proposed, how the proposals reflected the provisions of the relevant guidance documents, and the kind of design methods used. Furthermore, we report on the CHMP/SAWP positions on the proposed trial designs.

In Section Regulatory guidance documents, we give a brief review of the EMA and FDA guidance documents on adaptive designs (see Gaydos *et al*. [7] for a detailed discussion), which provide the basis for the assessment of specific study proposals. In Section Methods, the methodological background of our survey is presented. In Section Results, we report results of the survey and discuss several case studies. Our conclusions regarding the survey are presented in Section Discussion.

### Regulatory guidance documents

In the *Reflection Paper on Methodological Issues in Confirmatory Clinical Trials Planned with an Adaptive Design* (CHMP/EWP/2459/02) [3], a study design is defined as adaptive ‘if the statistical methodology allows the modification of a design element (for example, sample-size, randomization ratio, number of treatment arms) at an interim analysis with full control of the type I error’. The reflection paper emphasizes that the statistical principles for clinical trials outlined in the ICH E9 guidance document equally apply to adaptive designs. In particular, besides the control of the type I error rate (and the familywise type I error, in settings where several hypothesis are tested, see the *Points to Consider on Multiplicity Issues in Clinical Trials* [8]), it is required that ‘correct estimates and confidence intervals for the treatment effect are available’. An additional requirement specific to adaptive designs is that methods for the assessment of homogeneity of results from different stages are preplanned’. This is of special concern if the adaptations are based on unblinded interim data.

The reflection paper clearly states that interim analyses need to be justified. To prevent release of interim data, and thus to protect the integrity of the continuing trial, the interim analyses should ideally be performed by a sponsor-independent statistician who provides the analysis results to a sponsor-independent committee (as defined in the *Guideline on Data Monitoring Committees* [9]), which decides on the adaptation to be introduced. While it is required that the type of adaptations to be performed are preplanned in the trial protocol, the adaptation rule itself does not need to be fixed prospectively. The *Reflection Paper* concludes that, in a confirmatory phase III setting, the additional complexity of adaptive trials is only justified in ‘difficult experimental situations’ (as, for example, trials in small populations, see EMA Guideline on Clinical Trials in Small Populations [10]), and the reasons an adaptive design is proposed need to be described in detail.

The guidance document does not endorse specific statistical approaches for clinical trials with an adaptive interim analysis but formulates general minimal requirements that the methods need to satisfy. Several statistical methods have been developed that allow mid-trial design modifications in multistage designs that are based on unblinded interim data without compromising the type I error rate. Some approaches rely on detailed algorithmically pre-specified adaptation rules, while more flexible methods, as for example, adaptive combination tests [1] or the conditional error approach [2, 11] (which are closely related [12]), control the level without a detailed pre-specification of the adaptation rule.

While the EMA document focuses on the use of adaptive designs in the confirmatory setting, the FDA draft guidance *Adaptive Design Clinical Trials for Drugs and Biologics* [4], published in 2010, also discusses the use of adaptive designs in earlier phases of drug development and encourages their use in this setting. For confirmatory trials, the FDA document distinguishes between ‘well understood’ and ‘less well understood’ (due to limited experience of sponsors and regulators) adaptive designs. The latter category comprises mainly adaptive designs where adaptations are based on unblinded interim data. Similar to the EMA document, the FDA draft guideline states that type I error rate control is a primary statistical concern of a confirmatory adaptive clinical trial. In addition it is emphasized that unblinded interim analyses in a confirmatory settings raise concerns that access to unblinded data of persons involved in the conduct of the study may cause operational bias, thereby compromising the integrity of the trial. Overall the EMA and FDA documents share the same major principles.

## Methods

A text search of scientific advice letters issued between January, 1, 2007 and May 8, 2012 containing one of the following key terms: ‘adaptive design’; ‘flexible design’; ‘adaptive interim analysis’; ‘sample size reassessment’; ‘adaptive group sequential’ and ‘CHMP/EWP/2459/02’ (CHMP Reflection Paper on Methodological Issues in Confirmatory Clinical Trials Planned with an Adaptive Design) was performed. The resulting letters were reviewed and letters containing questions related to adaptive clinical trials in phases II or III were selected for further analysis. Based on the selected letters, details of the procedure (type of product, therapeutic area, rare disease, that is, a prevalence of <5/10,000, designation as orphan medicine, small- or medium-size enterprise status of the company (SME), year of the scientific advice, whether the study in question was a single pivotal study), the proposed adaptive study (type of primary endpoint, phase II or phase III study, initial number of study arms included, stopping for futility or efficacy, number of interim analyses, type of adaptations), and the CHMP/SAWP response were categorized.

We classified the CHMP/SAWP responses into three categories: ‘accepted’ for proposals where no major critical issue was identified and the adaptive trial proposal was endorsed; ‘accepted conditionally (concerns to be addressed)’ for procedures where the adaptive design proposal was endorsed in principle but critical issues were raised that need to be resolved before the trial can be endorsed; and ‘not accepted’ for proposals where CHMP/SAWP recommended a different experimental approach.

For the more recent procedures (1 January 2009 to 8 May 2012), additional details of the trial design were assessed. Note that the search strategy identified only scientific advice letters discussing clinical trials with design modifications at an interim analysis where at least one of the search terms occurred. Thus, the reported numbers give only a lower bound for the actual number of scientific advices given on adaptive designs. For example, an earlier review [13] identified 16 and 15 scientific advice letters on confirmatory adaptive designs for the years 2007 and 2008, respectively, using a different search strategy. Even though the absolute numbers of SA letters were different, the key findings were similar. Our research did not involve individual human data as the survey was restricted to official scientific advice texts on statistical methodology. According to the publication policy of the EMA, the manuscript was approved by the EMA review board.

## Results

### Characteristics of submitted studies

**Figure 1:**
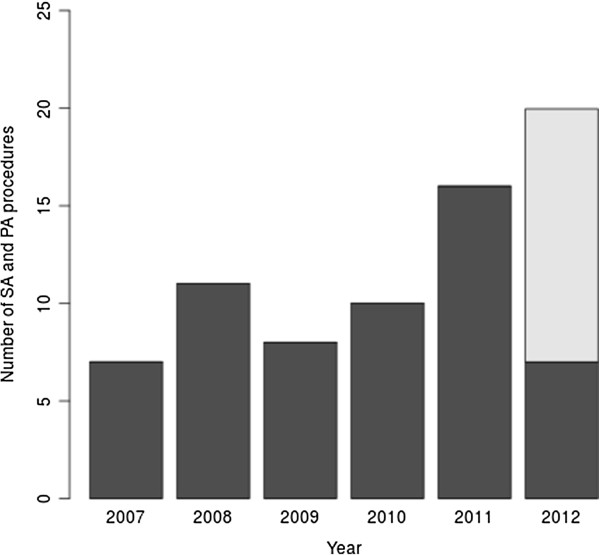
**Number of identified scientific advice (SA) and protocol assistance (PA) procedures per year.** The projection for 2012 (light gray) is based on linear extrapolation of the number of submissions received in 2012 until May 8, assuming the submission rate stays constant.

The text search identified 123 (83 from 1 January 2009 to 8 May 2012) scientific advice letters, of which 59 (41) addressed questions on an adaptive design for a phase II or phase III trial. Figure 1 shows the number of such procedures per year. There appears to be an increase in submissions in 2011 (note that the numbers for 2012 includes the data till May, 8 only), possibly an impact of the publication of the FDA guidance document in 2010. About two-thirds of the procedures were on clinical trials for new biologicals, new chemicals or advanced therapies, and the remaining were on known medicinal products. A total of 27 (46%) of the trials were performed in oncological indications, 35 (59%) on a rare disease, but only 21 (36%) had an orphan designation (see Table 1 for the descriptive statistics of all variables). A total of 15 (25%) of the requests for scientific advice were from small- and medium-sized enterprises. Almost all of the clinical trials (54, 92%) were planned as confirmatory trials and labelled as phase III or phase II/III studies. In 44 (75%) of the procedures, the adaptive clinical trial was proposed as the only pivotal study to be performed. A total of 30 out of these 44 proposals (68%) with a single pivotal trial were ‘rare diseases,’ and 16 (53%) out of those 30 actually had the orphan designation.

**Table 1:** **Descriptive Statistics of the scientific advice (SA)/protocol assistance (PA) procedures in the years 2007 to 2012**

Variable		***N (%)***
Number of SA/PA letters with questions on adaptive phase II, phase II/III or phase III designs		59 (100%)
Type of medicinal product	New chemical entity	23 (39%)
	Known chemical entity	13 (22%)
	New biological	13 (22%)
	Known biological	6 (10%)
	Advanced therapy	4 (7%)
Therapeutic area of the indication of the medicinal product	Infectious disorders	4 (7%)
	Oncology	27 (46%)
	Endocrine and metabolic disorders	3 (5%)
	Neurologic and psychiatric disorders	3 (5%)
	Cardiovascular	6 (10%)
	Diagnostics	1 (2%)
	Respiratory	3 (5%)
	Dermatology	2 (3%)
	Others	10 (17%)
Rare disease (prevalence of <5/10,000)		35 (59%)
Applied for orphan designation		21 (36%)
Small or medium enterprise		15 (25%)
Year when the SA/PA letter was issued	2007	7 (12%)
	2008	11 (19%)
	2009	8 (14%)
	2010	10 (17%)
	2011	16 (27%)
	2012	7 (12%)
Scale of measurement of the primary endpoint discussed	Time to event	28 (47%)
	Binary	20 (34%)
	Continuous	11 (19%)
Adaptive study is the only pivotal trial		44 (75%)
Development phase for which the adaptive clinical trial is proposed	Phase II or IIb	4 (7%)
	Phase II/III	16 (27%)
	Phase III	38 (64%)
	Pediatric study	1 (2%)
Number of arms of the adaptive trial discussed	1	2 (3%)
	2	34 (58%)
	3	15 (25%)
	>3	8 (14%)
Stopping for futility was planned for in the adaptive trial	Yes	31 (53%)
Stopping for efficacy was planned for in the adaptive trial	Yes	19 (32%)
Number of interim analyses planned in the adaptive trial	1	43 (73%)
	2	13 (22%)
	>2	3 (5%)
Type of adaptations planned (multiple answers possible)	Sample size reassessment	43 (73%)
	Population enrichment	5 (8%)
	Dropping of treatment arms	19 (32%)
	Other adaptations	4 (7%)
CHMP raised issues regarding type I error rate control		19 (32%)
Categorization of the CHMP advice regarding the adaptive study design	Accepted	15 (25%)
	Accepted conditionally (concerns to be addressed)	32 (54%)
	Not accepted	12 (20%)

Most sponsors (43, 73%) proposed trials with a single interim analysis. While a futility rule was discussed in the scientific advice letter for 31 (53%) trials, only 19 trials (32%) planned for early stopping for efficacy. The most frequently proposed adaptation was sample size reassessment (43, 73%), followed by dropping of treatment arms (19, 32%) and population enrichment (5, 8%).

In 17 (29%) of the 59 proposed adaptive trials, options to stop the trial in the interim analysis for both efficacy and futility were pre-planned; in 14 (24%), early stopping was allowed for futility only. For the 43 trials with sample size reassessment, the respective frequencies are 15 (35%) and 24 (56%). Furthermore, of the 43 trials with sample size reassessment, 7 (16%) additionally included the option to drop a treatment arm.

In the more recent procedures (41 procedures from 1 January 2009 to 8 May 2012), where additional items were assessed, see Table 2), we found that 33 (80%) trials were proposed with an unblinded interim analysis in which the proposed design adaptations are based on unblinded interim data. Interestingly, two of the studies included a blinded as well as an unblinded interim analysis. For the majority of studies, the interim analysis was planned to be performed by an external data safety monitoring board, an independent statistician or a contract research organization (25, 61%).

**Table 2:** **Additional variables, years 2009 to 2012**

Description		***N (%),***
Number of scientific advice (SA) and protocol assistance (PA) letters with questions regarding adaptive phase II or phase III designs		41 (100%)
Number of pivotal studies the company plans to conduct.	1	33 (80%)
	2	5 (12%)
	>2	1 (2%)
	No information	2 (5%)
Adaptive design was preplanned in advance of the conduct of the study	Yes	40 (98%)
	No	1 (2%)
The planned interim analyses will be performed in a blinded fashion	Unblinded	33 (80%)
	Blinded	3 (7%)
	No information	3 (7%)
	Blinded and unblinded	2 (5%)
Interim analyses are to be performed externally (for example, by an external data safety monitoring board, an independent statistician or a contract research organization)	No	5 (12%)
	Yes	25 (61%)
	No information	11 (27%)
Issues raised in the Committee for Human Medicinal Products (CHMP) answer regarding the proposed adaptive design (the issues raised relate both to designs that were conditionally accepted and those not accepted, multiple answers possible)	Adaptation strategy not sufficiently justified	12 (29%)
	Potentially biased results	12 (29%)
	Too many interim analyses	2 (5%)
	A single pivotal trial which is adaptive is not recommended	6 (15%)
	Control of type I error rate	12 (29%)
	Other issues	5 (12%)

#### Outcome of the Committee for Human Medicinal Products/Scientific Advice Working Party advice

While 12 (20%) of the 59 proposals for an adaptive clinical trial were not accepted, the great majority of the proposals were accepted (15, 25%) or conditionally accepted (32, 54%). In 19 (32%) of the advice letters the CHMP/SAWP raised issues regarding type I error rate control. In 12 (29%) of the more recent 41 procedures, the CHMP/SAWP raised concerns that the adaptation strategy was not sufficiently justified. In six (15%) of the advices it was stated that a single pivotal trial that is adaptive is not recommended for the development program considered. For 12 (29%), a concern regarding type I error control was raised, whereas in 12 (29%), it was stated that results could potentially be biased, for example, because of the selection of treatment arms. A too-large number of interim analyses were considered to be of concern in two of the procedures. Another common concern was that the extent of reflection and adaptation required at the end of phase II will be too extensive to make a phase II/III ‘seamless’ trial practical.

### Case studies

In this review, we present three illustrative examples on how adaptive designs were proposed by sponsors for real-case scenarios of planned pharmaceutical development program. All case studies presented here were discussed at scientific advice meetings between 2007 and 2012. We focus on the response of the CHMP/SAWP concerning the adaptive features of the proposed clinical trial designs: that is, the review is fully based on the SA answer letter the sponsor received at the end of the SA procedure. The case studies shall illustrate how a sponsor can interact early with regulators and how critical issues can be addressed.

#### Case study 1: sample size reassessment

The first case study concerns an open-label, two-armed, single pivotal phase III study of an anticancer drug for a rare disease. The study was planned to show superiority of the drug over a standard treatment for the primary endpoint of overall survival. The adaptive design was pre-planned and two interim analyses were intended to be performed by an independent data monitoring committee. The interim analyses were intended to be performed at 50% and 80% of events, given a fixed overall sample size.

At the second interim analysis, the sponsor planned to re-evaluate the targeted number of events and suggested an increase in the number of events by 20% if the interim results show a promising but not overwhelming trend. The event number was to be increased without increasing the sample size by extending the observation period. The decision to increase the number of events was to be made by an independent data monitoring board, which would receive unblinded interim data. Based on the interim results, the conditional probability to obtain a significant result at the end of the study was to be estimated under various assumptions on the survival distribution and effect sizes. In particular, a bootstrap-based method taking into account the empirical survival distribution function was considered. Furthermore, in both interim analyses, Haybittle-Peto stopping boundaries for early rejection of the null hypothesis were pre-planned. To control the type I error rate, the sponsor proposed to use the inverse normal method, which combines the data accrued before and after the interim analysis in a pre-specified way [14, 15]. The CHMP/SAWP stated that the design is acceptable from a statistical point of view if the type I error rate is controlled and operational bias is avoided. Based on concerns over the totality of evidence that would be available for a benefit-risk assessment, the CHMP/SAWP experts did not agree on the early stopping boundary to reject the null hypothesis in the first interim analysis, and stated that at the first interim analysis, the trial should be stopped for futility only.

For the analysis of the adaptive trial, the CHMP/SAWP requested a comparison of the treatment arms based on the standard fixed sample test statistics and that the sponsor performs the inverse normal test as a sensitivity analysis only. Compared to the fixed sample test, the inverse normal method down-weights the treatment effect observed in the second stage if the number of events is increased. If the sample size is increased only if a promising interim effect is observed, it has been shown that under suitable conditions the fixed sample test controls the type I error rate [16, 17]. In addition, in the application to survival data, the additional events will typically occur at later time points. Therefore, under alternative hypotheses where the survival curves initially separate but become closer at later time points, down-weighting the second stage test statistics (based mainly on late stage events) was considered undesirable. Note that an additional complexity of the proposed design, which was not explicitly discussed in the SA procedure, is the issue that sample size adaptations based on information of patients censored at the interim analysis may lead to an inflation of the type I error rate [18–20].

#### Case study 2: interim dose selection

The second case study is a proposal of seamless phase II/III designs for two pivotal placebo controlled superiority trials of a new chemical entity for the treatment of diabetic nephropathy, which were supposed to be conducted in parallel. The proposed primary efficacy parameter was a surrogate marker of kidney disease progression. The purpose of the adaptive design was to eliminate one of three initially tested dose strengths based on an interim analysis of the benefit/risk ratio in both trials. In this manner, the sponsor intended to seamlessly combine dose finding/refinement and pivotal testing for efficacy and safety. The interim analyses were pre-planned and to be performed by an independent data monitoring committee (IDMC) after 60% of 420 patients had completed 8 weeks of treatment (primary analysis for efficacy and safety planned for week 24, secondary analysis at week 52) in the first trial. At the time of the interim analysis, it was expected that in the second trial about 40% of patients would have been recruited. The decision on dose selection was to be performed based on interim data from both trials using pre-determined criteria for the primary efficacy and safety parameters. In addition, the interim analyses were intended for the identification of a single dose for a renal event outcome trial. In order to preserve trial integrity, the company proposed that the IDMC’s decision to eliminate one dose strength was to be implemented by a pre-created interactive voice response system to avoid communication of this information to the sponsor. However a dose recommendation for an additional renal outcome trial was to be communicated from the IDMC to the sponsor. With regard to the type I error rate, the sponsor proposed to use a Bonferroni adjustment to control the familywise error rate at a two-sided level of 5%. Because in the final analysis only two doses were to be tested and no early stopping of the study was foreseen, the sponsor suggested adjusting the level for two comparisons only and applying a two-sided Bonferroni adjusted level of 0.05/2 = 0.025 for each comparison. The statistical testing procedure was not endorsed, as it was not supposed to control the familywise type I error rate for the three hypotheses initially considered. Even though, in the final analysis, only two doses would be compared to placebo, these two doses were to be selected based on interim treatment effect estimates of three dose arms. This selection may lead to a biased test unless an appropriate adjustment is made. In this case, adaptive combination tests based on the closure principle [21–23] and adaptive Dunnett test [24] procedures based on the conditional error rate have been proposed by the CHMP/SAWP as valid methods. Furthermore, it was suggested that the sponsor should evaluate the advantage of the proposed design with respect to power and sample size compared to more standard design options as the improvement in efficiency may be small when taking into account the time needed for the conduct of interim analyses and decision making. Furthermore, it was questioned whether adequate safety evaluation would be possible to support dose selection at the proposed time of interim analysis. Finally, the communication of a single dose to be tested in the renal event outcome trial was considered as an overall risk to the integrity of the development program as it would have necessarily revealed some degree of information about the interim data.

#### Case study 3: post-hoc adaptations

The third case study was an open-label, two-armed, single pivotal phase III study of a new chemical drug for treatment of advanced ovarian cancer resistant or refractory to a defined chemotherapy. The study was planned to show superiority over an active control for the primary endpoint of progression-free survival (PFS) determined by an Independent Review Committee (IRC). Even though the study was an open label study, the sponsor had no access to randomization or treatment codes.

Although the active control was considered acceptable and had been licensed in the EU for the proposed indication, information on the effect size in the intended population was limited to small subgroups of patients. Thus, during the planning stage of the study, the sponsor had to determine sample size and power based on very limited data for estimated PFS of the active control. Estimated PFS for the test drug was based on results of a small phase II study in fewer than 30 patients. During the ongoing pivotal phase III study when patient accrual had been completed, external data from a well-designed study in the target population found PFS for the active control drug to be considerably longer (approximately 50%) than the estimate used in the planning phase. Furthermore, in accordance with the IDMC charter, the sponsor periodically reviewed aggregate blinded investigator-reported PFS data and found a median PFS significantly longer than the original expected median PFS estimate both for the active drug and the control.

In order to reduce the risk of a potentially underpowered study, the sponsor proposed to increase the number of IRC-determined PFS events for the final analysis from 254 events to 375 events based on the revised assumption on estimated longer PFS in the control group and preservation of the relative treatment effect by the active drug. The sponsor believed there was no impact on the type I error when performing the originally planned fixed sample test because the sample size adjustment was only based on blinded interim data and external information.

The CHMP/SAWP had no objections against the selected comparator in a superiority trial or the revised assumption regarding PFS. Furthermore, there was no concern regarding an inflation of the type I error for the final analysis. However, the CHMP/SAWP noted that the final estimates of the treatment effect might be affected by some (small) bias due to the sample size reassessment.

## Discussion

Adaptive clinical trials are a frequently considered design option for clinical trials proposed by sponsors who request scientific advice. Asking for scientific advice on innovative trial designs is an attractive way for sponsors to involve regulatory agencies already in the planning aspects of a clinical drug development program. In addition, a new option to discuss statistical methodology more broadly, and not specifically for a certain clinical trial, is the recently introduced EMA procedure for the ‘Qualification of novel methodologies for medicine development’ [25], which includes statistical methodology as well. The survey illustrates that for the majority of proposed studies, CHMP/SAWP gave an overall positive opinion albeit with critical comments. In general our review confirmed that scientific advices issued are in line with the CHMP reflection paper on adaptive designs. The CHMP/SAWP stressed on many occasions that in confirmatory clinical trials the number of adaptations and interim analyses should be kept to a minimum and will have to be thoroughly justified in each case.

However, even though a huge range of statistical methodology to avoid type I error inflation in adaptive clinical trials has been developed over the years, type I error control in adaptive clinical trials surprisingly is still a frequent major concern raised in the SA letters. For example, the testing strategy outlined in case study 2 (no multiplicity adjustment for the initially considered but dropped treatment arms) is a common but erroneous approach for multiarmed clinical trials with adaptive selection of treatment arms [26, 27]. Therefore, CHMP/SAWP’s refusal to endorse adaptive designs without strict type I error control is not based on a negative position towards adaptive designs per se but reflects that type I error control in confirmatory clinical trials is considered of key importance. Therefore, when discussing adaptive designs the sponsor should demonstrate that the statistical approaches chosen control the type I error.

A further issue raised in the scientific advice letters is the requirement for a sound justification for the planned adaptations. To justify why an adaptive clinical trial is a favorable design option, extensive simulation studies are typically required, where the operating characteristics of the adaptive design are compared to more classical approaches as fixed sample trials or several trials in sequence. While adaptive designs may increase the statistical efficiency, the additional complexities of such designs increase risks to the integrity of the trial. Therefore, a justification is required that demonstrates that potential advantages of the more complex design outweigh the risks.

An unexpected finding was that in a majority of the proposals the adaptive clinical trial is the only pivotal trial mentioned in the scientific advice request, but this raised concerns in only a few cases even though the EMA reflection paper on adaptive designs [3] in general does not endorse the application of adaptive designs in single pivotal trials. However, for orphan indications, the reflection paper notes that a single adaptive trial may be justified ‘if such an approach is more efficient to display the totality of available information’. Indeed, a large fraction of the submitted proposals focus on orphan indications. However, they also include other indications (oncology, in particular) where a single pivotal trial is the norm because a demonstration of superiority precludes further randomization against that comparator. Also, there are specific challenges to obtain sufficient information from standard phase II exploratory trials. The survey demonstrates that the scientific advice and protocol assistance process individually assesses proposals on a case-by-case basis where sponsors do not routinely follow the reflection paper. In the European regulatory system, the pivotal studies constitute the core of regulatory decision making for marketing authorizations, and the EMA reflection paper focuses on confirmatory adaptive designs. This may motivate sponsors to discuss the use of adaptive trial designs when used in later phases of drug development but not when they are used for internal decision making, as proof of concept or dose finding studies. Even though not explicitly mentioned in the reflection paper, the EMA generally also encourages the use of innovative methods in earlier stages of drug development. Besides the SA and PA procedures, the Agency provides nonproduct-related platforms such as Innovation Task Force Meetings, Scientific workshops and Qualification Procedures to discuss new methodological approaches. Sharing experience between sponsors and regulators also for exploratory adaptive trials can be of great value, not least because in some areas, exploratory trials may constitute the majority of adaptive trials performed.

## Conclusions

It is difficult to draw general inferences about regulatory standards and preferences from the case studies and the survey because the assessment by the SAWP and CHMP depends on the overall quality and the general context of the proposal (overall drug development program, type of medicinal product, indication, *etcetera*). However, there are a number of questions that are generally addressed by assessors when evaluating adaptive clinical trial proposals:

1. Is there a good rationale? Have alternative, more standard trial designs been considered?
2. Does the proposal fit well in the context of the development program and the data that will be available for the marketing authorization application?
3. Can the proposal be implemented without important damage to trial integrity?
4. Is the type I error rate controlled?
5. Has the potential bias of treatment effect estimates been evaluated?
6. Is the proposal practical and feasible?

Such questions are not specific for adaptive clinical trial designs but may also be asked of more conventional approaches. However, for adaptive designs, the assessment is typically more complex. Therefore, adaptive design proposals are regularly referred to EMA’s Biostatistics Working Party, which comprises statistical experts from the European regulatory expert network and also includes experts from academia. Regulators involved in scientific advice and protocol assistance procedures have to be aware of the latest scientific developments in statistical methodology and study designs when giving advice to sponsors.

## Authors’ information

Amelie Elsäßer is a statistician and worked for 5 years as a research assistant at the University Medical Center of the Johannes Gutenberg University Mainz, Germany. She conducted the work on this article while she was seconded from the University as National Expert to the European Medicines Agency in London from December 2011 to November 2012. Since October 2013 she has been Senior Statistician at Boehringer Ingelheim Pharma GmbH & Co. KG.

Martina Greco, Jan Regnstrom, and Thorsten Vetter are Scientific Officers at the EMA.

Marisa Papaluca-Amati is Head Scientific Support at the EMA.

Franz König is Associate Professor for Medical Statistics at the Medical University of Vienna and was seconded to the EMA from 2008 to 2010.

Robert James Hemmings is Statistics Unit Manager at the Medicines and Healthcare products Regulatory Agency, voting member of the EMA Committee for Medicinal Products for Human Use (CHMP), Chair of the CHMP’s Scientific Advice Working Party and member of CHMP’s Biostatistics working party.

Martin Posch is Professor for Medical Statistics at the Medical University of Vienna and was Scientific Administrator at the EMA from 2011 to 2012 and is observer of CHMP’s Biostatistics working party.

## Abbreviations

CHMP: Committee for Human Medicinal Products
EMA: European Medicines Agency
FDA: Food and Drug Administration
IDMC: Independent Data Monitoring Committee
IRC: Independent Review Committee
PA: protocol assistance
PFS: progression-free survival
SA: scientific advice
SAWP: Scientific Advice Working Party.
